# The German Medical Student and Physical Diagnosis

**Published:** 1884-11

**Authors:** 


### THE GERMAN MEDICAL STUDENT AND PHYSICAL DIAGNOSIS.

The medical schools of Germany rank so deservedly high that the desire naturally arises in us of the western world to know what manner of medical man is sent forth from these institutions of learning.

The key note of the whole matter was struck by a German physician, a graduate of the Friedrich Wilhelm University, at Berlin, but now practicing medicine in this country, when he gave us his impression of the difference between German and American doctors. As nearly as we can recall his words, he said: "In Germany, a physician must make a correct diagnosis, whether the patient gets well, or no; but, in this country, a practitioner does not seem to think it so necessary to make an accurate diagnosis, as to treat the patient to the best of his ability." This touches the very point of weakness in our system of medical instruction. German medical students are undeniably better prepared for their medical studies and more thoroughly drilled than are those in this country.

Favored with excellent instruction in all departments, they are particularly fortunate in the opportunities afforded them to become familiar with all methods and means of diagnosis. Not only is clinical material ample and completely under control, but the students are admirably prepared to utilize this material by a rigid course of instruction in normal and pathological anatomy. Moreover, instruction in diagnosis is not imparted chiefly by didactic lectures, or by illustration on the part of the teacher; but it is intensely practical. Every man is given an opportunity to use his hands, as well as his eyes and ears, and thus he acquires considerable manipulative skill before entering upon the actual practice of his profession. In all departments of the "Polyclinic," whether of surgery, internal medicine, of the eye an ear, throat, or gynecology, the patients are turned over to the students for examination, who are then required to report their diagnoses to the instructor. Referring to a period in Munich devoted almost exclusively to physical diagnosis, we desire in this connection to emphasize the value of percussion and ausculation to the general practitioner, under almost all circumstances, and the need of bestowing greater attention upon the attainment of marked proficiency in this direction. We can not do better than to narrate how physical diagnosis is taught in the capital of Bavaria. At the opening of each semester, Professor Joseph Bauer organizes classes in physical diagnosis, dividing them according to the experience of their members. These classes meet the professor four times a week, and are then conducted into the hospital wards, where they are assigned, in groups of three or more, to each patient, being expected to examine the case as carefully as possible and to report their diagnosis to Dr. Bauer upon his return, at the close of the hour. After hearing the opinion of each student, he then goes over the case minutely, pointing out its peculiarities in detail. The class of diseases thus examined are not confined to affections of the thoracic viscera, but embrace all conditions in which skillful percussion and ausculation may be required. Particular stress is laid upon the exact mapping out of the various organs, healthy or diseased. This is an excellent discipline, the students being spurred on by a double stimulus—desire to win the approbation of the professor, who is a diagnostician of acknowledged skill, and, on the other hand, a spirit of emulation between the students assigned to each case. Then, when the students advance a step higher and attend the medical clinics of Von Gietel or Von Ziemssen, their diagnostic skill is put to the test in a most admirable way. Von Gietel conducts his class into the wards, and selecting some "praktikant," as the men are called who take these practical courses, he requires him to examine a patient, explain his symptoms and suggest treatment, the student being expected thereafter to keep watch of the case. Ziemssen's method was different. Instead of giving his clinics in the wards, he has the patients wheeled on beds into the amphitheatre of the lecture room, within full view of all present. One of the "praktikanten" is then called down into the amphitheatre, and in conjunction with the master examines the patient, often having to endure a searching fire of questions. These quizzes are calculated to draw out all a man knows, and frequently summons the tell-tale blush of confessed ignorance into the scared face of some swaggering *medicus* who but a moment before looked puffed out with self-satisfaction. Ziemssen, whose name is so familiar to the medical profession of the civilized world, through his important contributions to medical knowledge in individual writings, and by his editorship of "Ziemssen's Cyclopedia of the Practice of Medicine," is a most happy clinical instructor, full of nervous activity, alive to every new discovery or suggestion in medicine, yet slow to adopt a novelty until having himself investigated and proven or disproved its merits. His delivery is easy and animated, while he is untiring in the illustration of his remarks, either upon the patient, the blackboard, or by plates and models. He is an adept in all means of diagnosis. He neglects nothing, however trivial, that can throw light upon the case. He is as equally at home with the microscope and the litmus paper, the sphygmograph and the thermometer, the laryngoscope and the tongue depressor. He examines critically all excretions, and never omits to note any phenomenon or deviation from the normal standard, however apparently insignificant. But he lays special stress upon percussion and auscultation as a means of diagnosis, and never loses an opportunity to impress upon his listeners the importance of always making a careful examination of the chest, no matter whether disease of the thoracic organs be suspected or no, for, as he says, "It will often throw unexpected light upon the case, and render clear and definite a diagnosis which was before shrouded in obscurity."

If, then, a man of Ziemssen's experience and professional attainments considers it always necessary to carefully investigate the condition of his patients' internal organs, how much more essential is it for one of less extended experience! And it is exactly in this regard, as it appears to us, that our general practitioners are careless, if not actually incompetent. How common it is for the physician, when called to see a new case, to content himself with a superficial examination! He feels the skin and pulse, takes the temperature, perhaps, looks at the tongue, inquires as to the symptoms, asks a few questions concerning the bowels and digestion, and then prescribes, contenting himself and the family with some vague diagnosis, wherein he confounds a symptom with the cause, or but too often saddles the whole thing on to the shoulders of that much-abused scapegoat, malaria. Why is this? Is it from indolence? from an underestimate of the importance of a thorough physical examination? or from an inability to make such an examination? Doubtless all of these reasons exist. But we think, as a rule, the last two really furnish the explanation of the too prevalent neglect in this country of adequate physical diagnosis. And, since that is so, the blame attaches not so much to the practitioner as to that system of medical training which failed to prepare him to appreciate the value of, and to skillfully employ, percussion and auscultation. How many institutions of medical instruction really afford their students adequate training in this direction? How many of the medical graduates go forth to practice, conscious of their ability to accurately diagnosticate diseases of the chest? Alas! but too few! Yet an assertion was made to us, last winter, by a physician of Chicago, to the effect that general practitioners have made such progress in the diagnosis of disease as to render specialists in diseases of the heart and lungs unnecessary. We daily learn of facts that teach another lesson. A lady recently complained because her physician, after making an examination over all her clothing, pronounced the apex of one lung to be indurated; whereas, it is needless to say, percussion of the bared chest brought out a normal vesicular resonance. In another instance, a practitioner mistook a superficial muscular dullness for high-pitched percussion note of pulmonary consolidation. We have in mind a case where the second stage of pneumonia was mistaken for a tuberculosis with the rapid disintegration of lung substance. Another, where the vicarious vesicular emphysema of a portion of a consolidated lung was mistaken for pneumo-thorax. And instances of inorganic cardiac lesions being pronounced organic are every-day occurrences. But why multiply examples? It is patent to candid minds that an improvement in this direction is called for. Our medical schools should adopt some system of teaching physical diagnosis which will compel every student to percuss and auscultate personally and not be satisfied with observing how it is done. And before being granted his certificate of proficiency, he should be compelled to give the instructor in this department practical demonstration of his ability to diagnosticate cases set before him. And we respectfully submit this matter to the attentive consideration of our medical colleges. To the busy practitioner, who cannot return to an institution for further instruction, yet who feels his incompetency to detect obscure morbid conditions within the thorax, to such we recommend that henceforth he embrace every opportunity to percuss and auscultate the chests of his patients, whether healthy or diseased, and he will soon be surprised to perceive the degree of keenness which his ear will acquire, while his percussing finger will detect minute differences in the resistance of parts percussed, of which he never before dreamed. He will thus often be enabled to clear up cases which before would have baffled his limited diagnostic powers, and will find a degree of success attending his therapeutic measures that will convince him how important accurate diagnosis is to a clear-sighted, rational, successful treatment.

